# BELMiner: adapting a rule-based relation extraction system to extract biological expression language statements from bio-medical literature evidence sentences

**DOI:** 10.1093/database/baw156

**Published:** 2017-02-26

**Authors:** K.E. Ravikumar, Majid Rastegar-Mojarad, Hongfang Liu

**Affiliations:** 1 Department of Health Sciences Research, Mayo Clinic, USA and; 2 Department of Health Informatics and Administration, University of Wisconsin-Milwaukee, Milwaukee, WI, USA

## Abstract

Extracting meaningful relationships with semantic significance from biomedical literature is often a challenging task. BioCreative V track4 challenge for the first time has organized a comprehensive shared task to test the robustness of the text-mining algorithms in extracting semantically meaningful assertions from the evidence statement in biomedical text. In this work, we tested the ability of a rule-based semantic parser to extract Biological Expression Language (BEL) statements from evidence sentences culled out of biomedical literature as part of BioCreative V Track4 challenge. The system achieved an overall best F-measure of 21.29% in extracting the complete BEL statement. For relation extraction, the system achieved an F-measure of 65.13% on test data set. Our system achieved the best performance in five of the six criteria that was adopted for evaluation by the task organizers. Lack of ability to derive semantic inferences, limitation in the rule sets to map the textual extractions to BEL function were some of the reasons for low performance in extracting the complete BEL statement. Post shared task we also evaluated the impact of differential NER components on the ability to extract BEL statements on the test data sets besides making a single change in the rule sets that translate relation extractions into a BEL statement. There is a marked improvement by over 20% in the overall performance of the BELMiner’s capability to extract BEL statement on the test set. The system is available as a REST-API at http://54.146.11.205:8484/BELXtractor/finder/

**Database URL:** http://54.146.11.205:8484/BELXtractor/finder/

## Introduction

Elucidation of biological pathway events involving drugs, proteins and diseases through extraction of knowledge from the scientific literature is one of the interesting challenges in biomedical text mining. The automatic extraction of such events will provide insights into the underlying molecular mechanisms of biological macro-molecular interactions and pharmacological dynamics. Despite multiple knowledge acquisition efforts to catalog biological events in databases, a considerable amount of knowledge is still buried in the scientific literature. Text mining offers the potential to bridge this gap and thereby overcome the amount of huge manual effort involved in database curation.

Although there has been significant text mining efforts in bio-medical domain addressing diverse problems (1) it is essential to have a common test dataset to benchmark their relative performance for fair comparison. BioCreative competition (2–9) has taken the lead in organizing such shared tasks. Biomedical text-mining community has also organized other shared tasks such as BioNLP shared tasks (10–12), and Drug-Drug interaction shared tasks (13). BioCreative shared task were being organized since 2006 and we recently had the fourth shared task organized in September 2015. Although the shared tasks substantially improved the state of the art of information extraction addressing diverse tasks none of the task addressed the issue of extracting normalized relations with entities and events mapped to standard representation. Biological expression Language (BEL) (33) attempts to formalize the knowledge expressed about various biological entities and events in a well-controlled vocabulary. In order to further improve the state of the art of information extraction, Rinaldi and colleagues (14) organized a shared task as part of BioCreative V shared task track4, which involves extraction of bio-medical relations from evidence statements from scientific literature texts and formalize the textual extraction in BEL representation.

As part of this shared task, we explored a rule-based approach to extract biological events from the sentences provided for the task and formalize them in BEL framework. We describe how we adapted an already existing rule-based information extraction system (15) for the task and discuss its performance in extracting BEL statements from evidence sentences provided for this task.

## Background

Text mining offers the potential to tap into knowledge hidden in the ever-increasing body of biomedical literature. Biomedical text-mining work may include simple retrieval and ranking/clustering of relevant literature (16, 17) from PubMed database, extraction of domain relevant entities (6), normalizing the entities to concepts in databases/ontologies (7), extraction of simple binary relations (4), extraction of complex relationships both within sentences and across sentences and formalizing extractions to semantic relationships (15, 18).

Term extraction and normalization is the first step towards extracting relationships from biomedical text. Term variation in biology is the first major hurdle for text processing., They often vary from simple orthographical variations (e.g. ERK-1, Erk1) to multiple synonyms (e.g. Erk-1, MAPK-3). Thanks to the existence of many terminological resources in the biological domain, especially curated ontologies and lexicons such as the Gene Ontology (GO) (19), Biothesaurus (20), BioLexicon (21) and UMLS (22), there is a way to overcome the term variation issue. Built around the above-mentioned lexical resources, various techniques (8) have been explored to recognize ontological concepts in an abstract/article. BioCreative I (3), II (5), III (7) and IV (9) and similar other shared tasks were organized for term extraction and normalization tasks, which resulted in significant improvement in the state of the art of entity recognition and normalization. Although the focus of the first two biocreative shared tasks were centered on gene normalization, subsequent competitions included entity normalization involving broader terms such as diseases, chemicals and other categories.

Extraction of relations between the entities/concepts becomes important once we identify the relevant concepts. Bio-medical event extract system systems employ different approaches such as rule-based (15, 23), statistical (24), grammar-based (25), supervised (26) or unsupervised learning approaches (27) to extract relations from biomedical texts. Both OpenDMAP framework developed by Hunter and colleagues and the ruled based system developed by Ravikumar et al., (15) are based on the broad framework of SemanticVerbNet (28). They define specific semantic frames to assign semantic role to the extracted arguments for a given predicate. OpenDMAP initially focused on specific event ‘biological transport’ though the notion was extended to handle protein–protein interactions. The rule based system proposed by Ravikumar et al., (15) has the capability to extract even complex relations involving complex events. On the other hand, Pustejosky et al., (24) used a robust statistical parser to extract ‘inhibit’ relations from biomedical text. Friedman et al., (25) employed a grammar based approach to extract broader set of relations. Most of the earlier works involved extraction of relations involving simple entities. Since the advent of shared tasks such as BioNLP and BioCreative, there has been thrust in the improvement of the state of the art of relation extraction beyond extracting simple relations. Notable among the work is the effort of Bjorne et al., that resulted in the development of TEES framework. TEES explored a supervised machine learning-based approach that took advantage of the grammatical structures due to syntactic parsing to extract complex relationships outlined in BioNLP 2009 and BioNLP 2013 shared tasks.

Although extraction of relationships is useful, the key challenge is to formalize the textual extractions in a representation that has both powerful syntax and expressive power to represent biological pathway/network semantics. We have standards for representing biological cascades/pathways such as Systems Biology Ontology (29, 30), Biological pathway exchange language (BioPAX) (31), Systems Biology Mark Up Language (SBML) (32), and Systems Biology Graphical Notation (SBGN) (33). There is an urgent need to have a mechanism to translate the textual assertions from scientific abstracts and articles to a standard representation. The distinct gap in the semantics between the assertions extracted from biomedical text and systems biology representation standards is the key bottleneck in this process. We ideally require a formalism that will enable us to bridge the gap in the semantics between text mining and systems biology. BEL has recently emerged as one such standard for representing qualitative causal relationships extracted from the biomedical text. BEL can serve as a link between the textual extractions and the quantitative pathway standards such as SBML, BioPAX, SBGN etc.

In this article, we describe BELMiner, a rule-based semantic parser that extracts biological event relationship across sentences to translate the textual extractions to BEL (34) statement as outlined in BioCreative Shared Task 2015 Track V (Fluck, Fluck). In the following sections we briefly describe our system, discuss the results and point out some of the limitations and how that resulted in certain kinds of errors. Finally we briefly describe our ongoing and future work, which may have the potential to improve the performance of the system and the state of the art in relation extraction and formalizing the textual extractions to formal representation.

## System description

**Figure 1:**
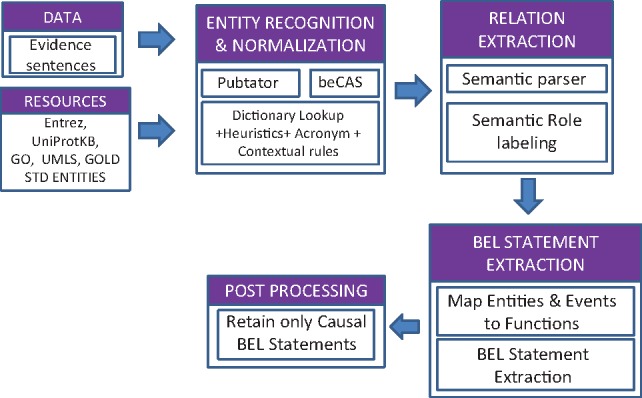
System architecture.

Figure 1 outlines the overall architecture of the information extraction system that we adapted for the BioCreative BEL task.

### Extraction of normalized entities

We used an ensemble of state of the art named entity normalization tools such as PubTator (35), beCAS (36) supplemented by a dictionary based lookup to ensure development of a high precision entity extraction and normalization system. For both PubTator and beCAS, we used the REST-API services provided by the respective tools. Besides Pubtator and beCAS, entity detection and normalization was supplemented with our own NER detection that employs a combination of approaches such as fuzzy dictionary lookup, heuristic rules and disambiguation based on contextual information.

### NER module

As a first step towards dictionary look up we compiled different dictionary from different sources such as Entrez (37), UniProtKB (38), GO (19), Comparative Toxicogenomics Database (39) and MeSH (43) to detect gene, biological processes, cell component, chemical names, and disease names respectively. Our internal NER has three features:

- Base noun phrases that lack morphological features. Consider a base noun phrase ‘bone morphogenetic protein-2’ that was not identified as a protein by both Pubtator and beCAS. The tokenization component translates the phrase to ‘bone morphogenetic protein 2’ due to which we found an exact match in Entrez and normalized to ‘p(HGNC:BMP2)’. Tokenization is applied to both the dictionary and the evidence sentences.
- Re-use of knowledge from acronyms: The entities mentioned in the evidence sentences may often contain only the short form of the protein, though the definition of the long form may be expressed in the abstract. We used the knowledge from the long form-short form pair association (15) to infer the semantic class of the entity. Consider an example phrase ‘inhibition of the AR by tolrestat’ where both Pubtator and BeCAS failed to identify ‘AR’ even as an entity. However, the long form ‘Aldose reductase’ was identified as protein and the acronym detection identified the long form short form association between ‘Aldose reductase’ and ‘AR’.
- Matching entities based on surface similarity of strings: Two entities with string similarity often belong to the same class. For example consider the two entities ‘Gi alpha(1) and Gi alpha(2)’ where Pubtator identified Gialpha(1) as protein, while it failed to identify ‘Gi alpha(2)’ as entity. The system not only successfully identified the entity based on string similarity comparison but normalized the entity to the right target name space p(HGNC:GNAI2).

We have priority rules, while resolving the conflict between multiple NER systems. For genes/proteins, chemical and disease names, we preferred the annotations of PubTator to that of beCAS. If PubTator failed to annotate genes, chemicals or diseases, we considered the consensus between beCAS and the dictionary based lookup. For detecting GO terms, we relied first on the annotations of beCAS, which was further supplemented by the dictionary lookup based on the vocabulary that we created from GO. For GO terms in addition to combination of beCAS and dictionary look up we also consider gene–GO term relationship to refine normalization. For example, consider the sentence ‘In the absence of CdCl2 pretreatment, ionizing radiation increased both expression and phosphorylation of c-Jun’. the phrase ‘ionizing radiation’ matched two phrases from GO ‘response to ionizing radiation’ and cellular response to ionizing radiation’ the latter being the child of the former. In such cases the system will identify the most specific one (‘cellular response to ionizing radiation’ in this case). However in the GO we have association between ‘JUN’ and ‘response to ionizing radiation’ and hence the algorithm prefers the parent over the child.

At times we also encounter situations where phrases match more than one dictionary source. In such situations, we have conflict resolution mechanisms. Consider the example sentence ‘FoxO1 protects beta cells against oxidative stress’ where the phrase ‘oxidative stress’ maps to two different dictionary sources MeSH ‘disorders’ and ‘GO’. Although the phrase exactly matches MeSH disorders dictionary, there is only a partial match with multiple GO terms such as ‘response to oxidative stress’, ‘cellular response to oxidative stress’ etc. The dictionary lookup during Phase 1 will prefer exact match over partial match. However during phase 2 we observed that gold standard annotators interpreted ‘beta cells against oxidative stress’ as a GO term ‘response to oxidative stress’. During Phase II the dictionary lookup against gold standard entity gets higher priority over normal dictionary lookup. Hence the phrase ‘oxidative stress’ is normalized to GO name space ‘response to oxidative stress’. If there are disagreements over the entity boundaries, we retained the longest match across different NER systems.

### Correction of named entity annotations with gold standard entities (phase 2 submission)

During phase 2, the organizers provided the gold standard for all the entities. We limited the dictionary lookup to only those entries that were provided in the gold standard. There were errors in the entity normalization pipeline. For example consider the phrase ‘**inhibition of calcium ionophore-induced ICAM-1 expression**’. Both PubTator and BeCAS identified ‘calcium’ as entity and normalized it as **ChEBI:‘calcium’**. However, in the gold standard annotation the ‘calcium ionophore’ was given as entity and normalized to **ChEBI:‘Calcium ionophore’**. Dictionary lookup against gold standard entity repository corrected the error in term extraction. Consider another example phrase ‘LPA-mediated mitogenesis’ the beCAS system normalized ‘mitogenesis’ to ‘positive regulation of cell proliferation’. Using the gold standard annotation we created a separate dictionary for GO terms in order to ensure that dictionary lookup normalizes ‘mitogenesis’ mention to ‘GOBP: “mitosis”’. This approach helped us to correct errors in the GO ontology terms, which was one of the weak link in named entity extraction and normalization pipeline.

### Extraction of biological events

**Figure 2:**
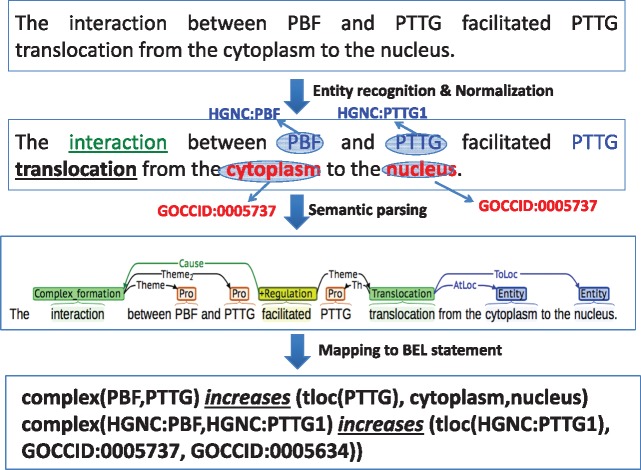
Illustration of individual steps of rule based semantic parser.

Figure 2 illustrates the individual steps of the overall system with an example. We used a rule-based semantic parser (15), which can handle discourse connectives, entity and event anaphora for effective synthesis and extraction of complete event information. The system besides identifying the syntactic arguments of verbs also assigns thematic roles. The frame-based semantic rule templates contain nearly 15 verb categories (including causal verbs) and >70 verbs and their inflections. The rules were developed by analyzing sentence structures pooled from different annotated corpora such as BioNLP shared tasks (10–12) and the one used in other studies (15). The semantic parser begins as a linear parse, but builds upon its linear structure to handle complex recursive grammatical structures such as appositions, selective prepositional phrase attachment, and co-ordinations (involving entities, events and clausal). It highly depends on semantic information while linking events across clausal boundaries. Besides, the system also handles anaphora resolution at both the entity and event levels. Due to space constraints we briefly describe some of the rules with examples. Three different rules given below apply on the example sentence shown in Figure 2 to extract single BEL statement.

- Rule 1: [**BindingEvent**_NP_] between [Entity 1_NP_](, [Entity 2_NP_])* and [Entity N_NP_]
- Rule 2: [Entity 1_NP_] [**TransportEvent**_NP_] from [Location_NP_] to [Location_NP_]
- Rule 3: Rule1 [**RegulateEvent**_VP_] Rule2

Rule 1 extract the physical interaction that happen between two or more entities and Rule 2 extract the movement of protein from one subcellular location to another. Rule 3 connects two sequential events extracted by the earlier rules. Although the first two rules involve extraction of relationships involving entities, the third rule links these two events thereby help extract the complete BEL statement.

*Extraction of relations across clausal boundaries.* The semantic parser has features to link events across clausal boundaries. Consider the following sentence: ‘“Tip60” and the transcriptional repressor “ZEB (zinc finger E box binding protein) interact” specifically in the yeast two-hybrid system “and” additively “inhibit the CD4” enhancer/promoter activity in Jurkat cells’. In the first pass the system extracts simple relations involving entities. For example it extracts ‘Tip60’ (HGNC:KAT5) and ‘ZEB’ (HGNC:ZEB1) as subject of the verb ‘interact’. The verb ‘interact’ belongs to ‘BindingEvent’ semantic class leading to extraction of ‘complex(Tip60, SEB)’ as the inferred object. Similarly the system extracts ‘CD4’ as the object for the verb ‘inhibit’ though the subject is empty. The co-ordination rule identifies that the verb ‘inhibit’ is in co-ordination with the verb ‘inhibit’. The rule extracts event as the subject for the verb ‘inhibit’ paving way for extraction of complete BEL statement.

*Text simplification.* BELMiner has some sentence simplification component where certain linguistic structures such as appositions were masked to extract relations across clausal boundaries. For example consider the sentence ‘The **vasoactive intestinal peptide (VIP)** and the **pituitary adenylate cyclase-activating polypeptide (PACAP)**, *two immunomodulatory neuropeptides that affect both innate and acquired immunity*, ***down-regulate* IL-12 p40** and **inducible NO synthase** expression in LPS/IFN-gamma-stimulated macrophages’. The sentence simplification identified the apposition (in italics and underline) and masks the structure. A simple rule ‘**[Entity_NP_] [Negative Regulation_VP_] [Entity_NP_]’**extracts four BEL statements from the simplified sentence: ‘The**VIP** and the **PACAP*down-regulate* IL-12 p40** and **inducible NO synthase** expression in LPS/IFN-gamma-stimulated macrophages’. The entity in the above rule may be either an entity within a simple base noun phrase or compound entities in coordinated noun phrases as shown in the above example.

### Mapping semantic parser output to BEL annotations

Mapping the extraction output of semantic parser was done at two levels. The rules to translate textual extractions into BEL statement were inspired by the work of Fluck et al. (2013) (40). (i) Mapping certain biological events of the semantic parser to BEL functions. (ii) Mapping causal relations (decreases, increases, directlyIncreases and directlyDecreases) that connect BEL functions to complete BEL statements. Table 1 lists some of the examples of how we map the NLP system extractions to BEL functions.

**Table 1.** Mapping NLP system output to BEL functions

Event/Entities	BEL function
phosphorylation of **PDE3B** on **serine-273**	p(HGNC:PDE3B,pmod(P,S,273))
translocation of **HSF1**	tloc(p(HGNC:HSF1))
expression of **ICAM-1**	act(p(HGNC:ICAM1))
truncal **obesity**	path(MESHD:Obesity)
interaction of **cyclin A1** with **E2F-1**	complex(p(HGNC:CCNA1),p(HGNC:E2F1))
**cyclin A1** was complexed with **CDK2**	complex(p(HGNC:CCNA1),p(HGNC:CDK2))
**glycerol kinase** enzymatic activity	act(p(HGNC:GK))
activates STAT3	increases act(p(HGNC:STAT3))

There are certain events extracted by the system that could not be directly translated to BEL functions. For example consider the example sentence (SEN: 10000052; 10409724) ‘**Signaling by the IL-6 receptor** is mediated through the signal transducing subunit gp130 and involves the **activation of Janus-associated kinases, signal transducer and activator of transcription 3 (STAT3), and mitogen-activated protein (MAP) kinase’**. The system extracts ‘signaling(HGNC:IL6-R)’ activates (HGNC:STAT3) as one of the relations, where the ‘signaling (HGNC:IL6-R)’ doesn’t translate to any BEL function. Such functions are dropped from the final BEL statement and further simplified to p(HGNC:IL6-R), which is consistent with BEL syntax.

Most of the complete BEL statements are causal relationship between two simple functions or complex functions characterized by one of the four classes of verbs as outlined in Table 2. The algorithm maps the extracted entities or events between individual entities to BEL functions. Some of the examples for translating textual extractions to BEL functions are shown in Table 1. We generate BEL statement connecting two BEL functions linked by certain classes of verbs, which broadly fall into the four categories mentioned in Table 2. For example from the sentence: ‘**FcgammaRIIB** inhibits intracellular signaling upon ligation of IgG-immune complexes, and can *suppress***inflammation** and **autoimmunity.’**, the system extracts the relations decrease between the protein ‘**FcgammaRIIB**’ **(p(HGNC:FCGR2B))** and two diseases ‘**inflammation’(path(MESHD:Inflammation))** and ‘**autoimmunity’(path(MESHD:‘Autoimmune Diseases’))**. Once the individual entities are mapped to simple functions as shown in the parenthesis, the relation extracted by the co-ordination clausal rule (40) of the system. For the verb ‘suppress’ verb the simple functions were identified as arguments, while the verb ‘suppress’ is mapped to ‘decreases’ class. This results in the extraction of two BEL statements due to co-ordination: **p(HGNC:FCGR2B) -| path(MESHD:‘Autoimmune Diseases’)** and **p(HGNC:FCGR2B) -| path(MESHD:Inflammation)**. Consider another example phrase ‘**AKAP220** fragment is a competitive *inhibitor* of **PP1c***activity*’. In this sentence the phrase ‘**PP1C activity**’ is extracted as ‘activity(PP1C)’, which is further mapped to a BEL function ‘**act(p(HGNC:PPP1CC))’**, while ‘**AKAP220**’ is mapped to ‘**p(HGNC:AKAP11)**’. The system extracts the BEL statement ‘**p(HGNC:AKAP11)** decreases **act(p(HGNC:PPP1CC))’**.

**Table 2.** Verbs for causal relations

S. No	Verb categories	Verbs
1	decreases	reduce, decrease, suppress, block, down-regulate, decrease, down-regulation, inhibit
2	increases	increase, induce, activate, enhance, up-regulate, up-regulation
3	directlyIncreases	increase verbs preceded by an adjective “directly”
4	directlyDecreases	decrease verbs preceded by an adjective “directly”

### Filtering irrelevant annotations

After extracting the complete BEL statements we filter out BEL statements, that do not contain four classes of causal verbs namely, increase, decrease, directly increases or directly decrease. For example consider the sentence ‘**endoG** represents a **caspase-independent apoptotic pathway**’ where the NLP system extracts ‘**p(MGI:Endog)***represents* bp(**caspase-independent apoptotic pathway)**’. Since ‘**caspase-independent apoptotic pathway**’ could not be mapped to any BEL namespace it is replaced with bp(PH:Placeholder). However, the verb ‘represent’ do not fall into any of the four major causal verbs. Hence the BEL statement is dropped altogether from the final extraction. We made two submissions for the BEL task. Whenever we did not have high confidence in a namespace we replaced them with PH:placeholder so that they were not considered as precision error. In the first submission (Run1 in Table 3), we retained BEL statements that contains PH:Placeholder while in the second submission (Run2 in Table 3), we filtered all the statements containing the placeholder statements altogether.

**Table 3.** Performance of BELMiner on BioCreative BEL task (with and without gold standard entities)

Class		Entities from gold standard			Entities from NER		
		Pre (%)	Rec (%)	F-mes (%)	Pre (%)	Rec (%)	F-mes (%)
Term (T)	Run1	91.8	74.67	82.35	82.03	59.33	68.86
	Run2	92.51	70.00	79.70	83.33	50.00	62.5
FS	Run1	51.47	62.50	56.45	50.77	58.93	54.55
	Run2	51.61	57.14	54.24	54.72	51.79	53.21
Function	Run1	25.53	36.36	30.00	27.78	37.88	32.05
	Run2	27.06	34.85	30.46	30.67	34.85	32.62
Relation-Secondary (RS)	Run1	**87.71**	**77.72**	**82.41**	**76.84**	**67.33**	**71.77**
	Run2	**94.38**	**74.75**	**83.43**	**92.37**	**59.9**	**72.67**
Relation	Run1	**77.93**	**55.94**	**65.13**	**69.37**	**38.12**	**49.20**
	Run2	**77.93**	**55.94**	**65.13**	**69.37**	**38.12**	**49.20**
Statement	Run1	32.09	21.29	25.60	26.42	13.86	18.18
	Run2	32.09	21.29	25.60	26.42	13.86	18.18

Pre, precision; Rec, recall; F-mes, F-measure.

## Results and discussion

In the BioCreative V BEL task, the evaluation was carried at five different levels namely, Term-Level, Function-Level, Relationship-Level, Full Statement and Overall Evaluation. It was further carried out in two phases (i) without gold standard named entities and (ii) after providing the gold standard entities. For more description of the task and the BEL annotation kindly refer to the task description of Rinaldi et al. (2016)(14) and Fluck et al. (2016) (41).

Our team participated in both phases and for all evaluation except the term level. Table 3 outlines the results of the system for both runs of the system with and without the gold standard entities, respectively. The system was evaluated on standard metrics namely Precision, Recall and F-measure.

From Table 3, we can infer that the performance of the NLP system (row 6 of Table 3) in extracting a complete BEL statement is very low. Using gold standard entity instead of our ensemble of NER system resulted in significant improvement in the overall F-measure (nearly 7%). Very low performance of BEL statement extraction is not surprising given that the performance of the system in extracting the BEL function (row 3 of Table 3) is only 32%. Mapping textual extractions to BEL function is the performance-limiting step of our NLP system. The performance of Function-Secondary (FS) extraction is higher in mid 50%, which may be due to the reason that the system is capable of correctly extracting simpler functions involving entities, while its ability to extract recursive (or) complex functions is limited. On the other hand, the NLP system performs well in identifying the core relation, which is very evident from the rows 4 and 5 of Table 3. Being a rule-based system, the precision is reasonably higher (in the mid-70s to 80%) with a reasonable recall. In the Phase 2 evaluation, the performance of relation extraction (Row 4, Column3 of Table 3) is even higher with a significant gain of nearly 13% over phase 1. From the above results, we can infer that the entity recognition and normalization did have a positive influence in correctly extracting the BEL statement.

Five teams participated in the task 1 in both phases. In the phase I, our system achieved highest performance in 5 out of the 6 evaluation criteria. Our ensemble NER outperformed most of the systems in this stage (where gold standard entities were not provided) by >10% in terms of F-measure. The magnitude of difference in the function, secondary function and relation extraction criteria were even greater where our system outperformed the next best system by nearly 20% in F-measure. We lagged behind couple of systems in the complete BEL statement extraction. This is primarily due to the fact that we paid too much attention in improving the extraction of the elements of BEL statements. We believed that this would eventually improve the performance of BEL statement extraction. Few programming errors and with marginal fine-tuning our system would have performed well in the task. The trends were exactly similar even during the phase2 evaluation. Table 4 shows that the results significantly improved due to a single change in our rule while translating the relations to a formal BEL statement. Instead of mapping ‘expression of PROTEIN’ to ‘ tscript(p(PROTEIN NAMESPACE))’ we changed the rule to map to p(PROTEIN NAMESPACE). The results in Table 4 did not use any of the information provided in the gold standard. The overall F-Measure for BEL statement increased from 18.8 to 39.2%.

**Table 4.** Performance of BELMiner on BioCreative BEL task (Post Shared Task Improvements)

Class/Run2		Entities from NER		
		Pre (%)	Rec (%)	F-mes (%)
Term (T)	Run2	83.89	50.33	62.92
Function secondary (FS)	Run2	85.19	41.07	55.42
Function	Run2	71.43	30.3	42.55
Relation-Secondary (RS)	Run2	93.13	60.4	73.27
Relation	Run2	69.37	38.12	49.20
Statement	Run2	59.6	29.21	39.2

Pre, precision; Rec, recall; F-mes, F-measure.

### Impact of NER components on BEL statement extraction

**Figure 3:**
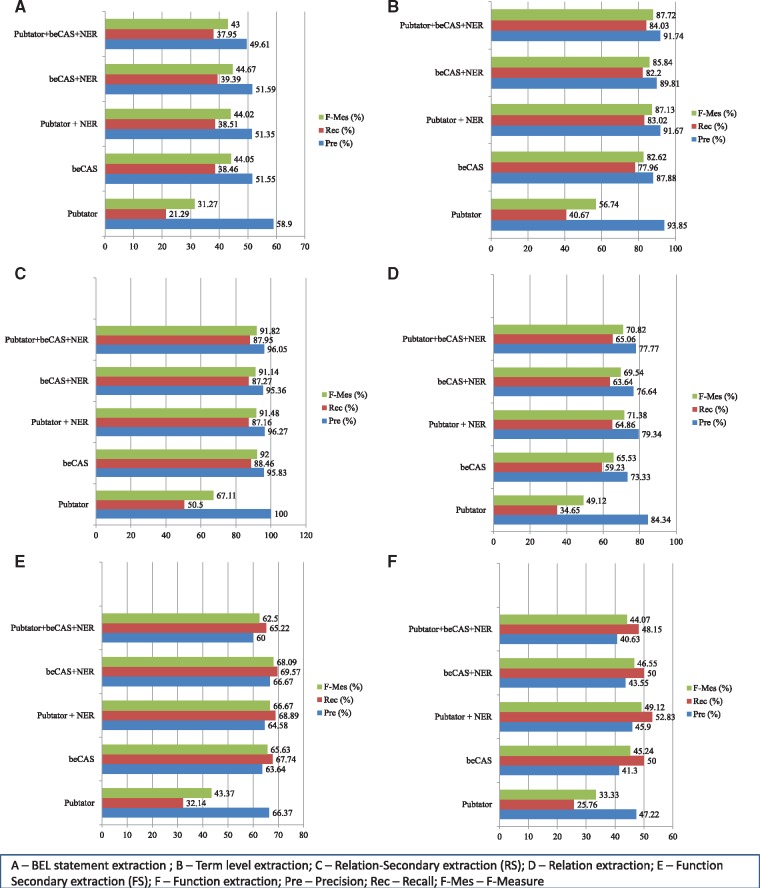
Impact of different NER components of BELMiner on BioCreative BEL task.

We evaluated the differential impact of various NER components of the system on five different criteria outlined for the competition on the test data set. For this evaluation we considered only the complete BEL statement. Figure 3 outlines the performance of various components of BELMiner on the test data of first task of BioCreative V Shared task. We evaluated the performance of five different systems. In two systems we used two external NER systems Pubtator and beCAS as standalone system. We also evaluated the performance of PubTator and beCAS in combination with in-house developed NER functionalities, which act complementary to the two independent systems. We also used the ensemble system similar to Run2 (in Table 3) that we used for the task. We included the one change in the final step of translating textual extraction to BEL statement we discussed towards the end of Results and discussion. For the competition we mapped ‘expression of PROTEIN’ to ‘tscript(p(PROTEIN NAMESPACE))’. We found this error to be a major source of false positives in our BEL statement extraction. In the current submission we modified the translation to ‘p(PROTEIN NAMESPACE)’.

*Term extraction.* Figure 3A outlines the impact of different NER systems on the extraction of complete BEL statement from the evidence sentences. Figure 3B shows the performance of various NER components on term extraction. Although Figure 3C and 3D lists the performance of five systems on the extraction of secondary relation and primary relation, Figure 3E and 3F outlines the secondary function and function level extraction performance. All the systems achieved relatively higher precision for term extraction. Except for standalone Pubtator most other systems achieved very high recall. Although Pubtator does not extract other entity classes such as ‘biological processes’ etc. that alone do not contribute to the lower recall. Our analysis revealed that even among protein classes it failed to extract nearly 15% of the entities. The standalone NER module compensated for the limitations of the PubTator as we observe a substantial increase in recall with very marginal decline in the precision of term extraction when we combined these two systems (PubTator + NER in Figure 3B). The new experiments revealed that the standalone beCAS system achieved very high performance on most of the tasks including term extraction. For term extraction the ensemble system achieved the highest performance in terms of F-Measure (87.72).

*Secondary relation and relation extraction.* The trends were almost similar for secondary level relation extraction to the term extraction (Figure 3C). Standalone beCAS achieved the highest performance in terms of F-measure for secondary relation extraction. Though trends for relation extraction continued, we observed nearly 20% points decline (Figure 3D). The major cause of this decline is the inability of the system to extract long distance dependency relations across clausal boundaries. Lack of semantic inference contributed to this decline.

*Secondary function and function extraction.* New analysis re-confirmed that extraction of BEL functions is the major limiting factor in BEL statement extraction. However we observed a marginal increase in the extraction of both secondary and primary BEL functions when compared with the results that we obtained during task submission (Figure 3E and F. A single correction of mapping ‘expression of PROTEIN’ to protein abundance instead of activity of protein abundance alone improved the performance of the system by 10% in the extraction of secondary functions and function.

*Net impact on BEL statement extraction.* The gain in the performance of BEL functions has visible impact on the complete BEL statement. We observed nearly a jump by nearly 20% (beCAS + NER in Figure 3A) in extracting BEL statements. Building upon the improved performance of NER and simple corrections alone contributed significantly to the improvement of BEL statement extraction. The results that BELMiner achieved should be treated as the upper boundary what the rule based system can reach if maximum number of terms and functions can be annotated automatically.

### Error analysis

We did a brief error analysis to understand the reasons behind the low performance of BEL statement extraction. We recognized problem at three levels. (i) Relation extraction, (ii) Identifying BEL functions, (iii) Term extraction and (iv) Miscellaneous errors such as programming errors. *Errors due to relation extraction.* (i) Long distance relation extraction, (ii) Lack of ability to draw semantic inferences, (iii) Lack of formal representation of semantic parser rules and (iv) Lack of predicate definition in the semantic parser vocabulary are some of the reasons causes for lower relation extraction performance of relation extraction which in turn impacted the performance of BEL extraction.

The semantic parser component of BELMiner failed to extract relations beyond clausal boundaries especially while handling co-ordination clauses. Consider the following sentence: ‘The inducible expression of ANG promoter appears to be mediated by **physical association** of *p300 with STAT 5B in liver***and***STAT 3 and STAT 5A in heart.*’. The system fails to recognize the co-ordination between two prepositional phrases ‘*p300 with STAT 5B in liver’* and ‘*STAT 3 and STAT 5A in heart’* while it recognizes the binding event (characterized by the phrase ‘physical association’) between p300 and STAT5B it fails to recognize the second binding between STAT3 and STAt5A leading to recall error.

BELMiner do not have capabilities to draw both logical and semantic inferences often expressed by the author while describing biological events. For example consider the sentence ‘More importantly, the **Dnmt1***knockdown* blocked the methionine-induced **reelin** and **GAD67 mRNA***down-regulation*.’ The author uses double negation to indicate a positive regulation event, which the system failed to detect leading to false positives. Knocking down the protein ‘Dnmt1’ blocks an event indicate that the protein ‘Dnmt1’ actually facilitates the event. The system instead of extracting ‘p(HGNC:DNMT1) -> a(CHEBI: methionine) -> p(HGNC:RELN)’ extracts a conflicting relation ‘p(HGNC:DNMT1) -| a(CHEBI:methionine) -> p(HGNC: RELN)’. The rules in the semantic parser to extract events from the biomedical text are not represented using any standard ontological definitions. For example consider an example sentence ‘**P21-activated protein kinase gamma-PAK** (Pak2, PAK I) is **cleaved** by **CPP32**’ for which the system extracts ‘**p(HGNC:CASP3) decreases act(p(HGNC: PAK2))**’ as the BEL statement. The semantic parser of BELMiner classifies the verb ‘cleave’ under protein activity disruption category. Although mapping the verb ‘cleave’ to BEL statements it is considered as event of negative regulation class. By ontologically representing interaction terms such as ‘clevage’, it is possible to solve issues such as the one encountered by BELMiner. Besides the entities ‘CPP2’ function and ‘PAK2’ are mapped to ‘p(HGNC:CASP3)’ and ‘act(p(HGNC:PAK2))’, respectively. The gold standard BEL annotation BEL ‘**cat(p (HGNC: CASP3)) directlyIncreases kin(p(HGNC:PAK2))’** infers that the kinase activity of the protein is diminished.

For example consider the phrase ‘endoG ***represents*** a caspase-independent apoptotic pathway initiated from the mitochondria’. The semantic parser do not consider the verb ‘represents’ does not fall into any of the predicate classes (such as regulation or causal etc.) that the semantic parser targets to extract relation. Hence the system did not extract any BEL statement for the sentence.

*Named entity errors.* Named entity recognition and normalization especially identification and normalization of GO terms also contributed to certain errors. In the phrase ‘FoxO1 protects beta cells against **oxidative stress** by forming a complex with the promyelocytic leukemia protein Pml …’, where ‘oxidative stress’ is mis-identified as disease ‘path(MESHD:Oxidative stress) while in the gold standard annotation it is identified as biological process bp(GOBP: “response to oxidative stress”)’. Unless we use external knowledge such as gold standard annotation it is difficult to infer the right semantic class of the phrase. The term extraction does not have capability to handle lexical variants, which also contributed to recall errors. Consider the example phrase ‘**endoG** represents a caspase-independent apoptotic pathway initiated from the mitochondria’. This example was also discussed under errors due to relation extraction in the earlier section. The system also failed to recognize ‘apoptosis’ as GO biological process term. The system does not have the capability to handle such lexical variations. Stemming or handling lexical variants using resource like BioLexicon could help address this issue systematically.

*Programming errors.* We observed errors due to some programming errors. For the phrase ‘anti-apoptotic protein Bcl-x(L) closes VDAC by binding to it directly’ the system extracted ‘**complex(p(:),p(HGNC:BCL2L1)) -| act(p (HGNC: VDAC1))’** as BEL statement. Due to programming error the system extracted empty function ‘p(:)’ instead of ‘**p(HGNC: VDAC1))’** that leads to both precision and recall error. We fixed all such minor programming errors in our latest evaluation, which reflects in the improved performance.

## Conclusion

In this work, we discussed the challenges of a rule-based information extraction system to BioCreative BEL extraction task. The system though achieved very low performance in BEL statement extraction and function detection; it achieved a very balanced performance in the relation extraction task. Lack of rule sets to map textual extraction to BEL formalism, named entity recognition and normalization, lack of methods to infer deeper biological semantics were some of the main reasons for lower performance of BEL statement extraction. With some fine-tuning, we believe that we can address some of the errors of this system to further improve the performance of the system.

## Limitations and future work

The system being a rule-based though had several advantages but suffers from rapid scaling. The amount of manual effort and time required to fine tune the system is enormous when compared with a machine learning approach. The detection of GO terms especially the biological processes and Molecular Function has been one of the weak links in the NER system. This has impacted the performance of the BEL extraction significantly. In order to address this shortcoming, we plan to create an ensemble exclusively for GO terms and supplement the existing state of art system with heuristics. The event ontology that we use for relation extraction lacks a formal framework. We believe that adopting formal ontological framework such as Interaction Ontology (INO) (42), will provide a more formal approach to design and model relations extracted from biomedical texts. We strongly believe that the current semantic frames defined for rule extraction can be easily migrated into INO kind of framework, which will better enable to draw inferences based on the semantic class of the predicate. In parallel, we also plan to explore machine learning approach for specifically extracting BEL statements and relations from biomedical text in general. Currently, we are creating guidelines to annotate instance level annotations for entities, relations and BEL statements for the entire BEL corpus released in track4. We have already completed annotation of entire sample set and small portions of training set. We are currently revising guidelines based on the initial IAA to further continue the annotation. We hope to create a text level annotation for every BEL statement provided for this task. We plan to use this corpus to further train a machine learning approach for entity normalization and BEL statement extraction. Although we intend to adopt standard framework such as INO to define and model rules, we believe that rule-based systems may not scale well for different problems. Machine learning models have the inherent capability to learn from prior labeled corpus. The performance of a machine learning system depends on the amount of training data. We may require additional rules and heuristics to achieve BEL statement extraction performance to the desirable levels. We strongly believe that hybrid architectures incorporating both rule based and machine learning based approaches not only has the potential to scale but perform well in most of the problems.
